# Social-emotional and behavioural problems in young children of healthcare worker mothers during the COVID-19 outbreak: a case-control study

**DOI:** 10.1186/s12888-024-05801-4

**Published:** 2024-05-30

**Authors:** Mahmut Caner Us, Perran Boran, Sıddika Songül Yalçın, Refia Gözdenur Savcı, Bahar Kural, Dilşad Foto Özdemir

**Affiliations:** 1 Institute of Health Sciences, Social Pediatrics PhD Program, Marmara University, Istanbul, Turkey; 2 School of Medicine, Department of Social Paediatrics, Marmara University, Istanbul, Turkey; 3 Faculty of Medicine, Department of Pediatrics, Hacettepe University, Samanpazari, Ankara, 06100 Turkey; 4 Department of Pediatrics, Alaçam State Hospital, Samsun, Turkey; 5 School of Medicine, Department of Pediatrics, Istanbul Haliç University, Istanbul, Turkey; 6 School of Medicine, Department of Child Psychiatry, Hacettepe University, Ankara, Turkey

**Keywords:** Toddlers, Pandemic, Healthcare workers, Socio-emotional development, Sleep

## Abstract

**Background:**

The pandemic has had a significant impact on the daily lives of children and their families, particularly the children of health care workers, due to changes in family routines as a result of their parents’ work schedules. We aimed to explore the socioemotional and behavioural (SEB) problems of children of healthcare worker mothers (HCWM) during the COVID-19 pandemic and compare them with age-matched children and their mothers from other occupations.

**Method:**

A case-control study design was applied, and a snowball approach was used to enrol volunteered participants aged between 6 and 36 months of age, through a Google survey. We used the Brief Infant-Toddler Social and Emotional Assessment (BITSEA) questionnaire to assess children’s SEB problems and a Brief Symptom Inventory (BSI) to evaluate the psychological distress of mothers. Differences in BITSEA, BSI and MSPSS scores were examined using chi-square and Mann-Whitney U tests, as appropriate. A logistic regression model was used to identify independent predictors of children’s behavioural and emotional problems.

**Results:**

In total, 600 questionnaires were analysed. It was observed that children in the HCWM group were separated from their mothers more often and for longer periods of time than their counterparts (*p* < 0.010, *p* = 0.002). Changes in the child’s structured outdoor activities during the pandemic period were more likely to be observed in the HCWM group (*p* < 0.05). The percentage of children with the BITSEA problem subscale above the subclinical cut-off, externalizing and dysregulation scores were significantly higher in the HCWM group (*p* = 0.044, *p* = 0.031, and *p* = 0.016). Moreover, each point increase in BSI global index scores (*p* < 0.001, RR:3.34, 95%CI:1.91–5.82) was found as a risk factor for clinically significant SEB problems.

**Conclusion:**

Overall, the current study suggests HCWM’s have experienced occupational inequality, and young children of HCWM’s were at increased risk for externalizing and dysregulation problems during the pandemic. Maternal psychological stress had a significant impact on their children’s socio-emotional well-being.

**Supplementary Information:**

The online version contains supplementary material available at 10.1186/s12888-024-05801-4.

## Introduction

The COVID-19 epidemic began in China, spread quickly to other countries, and has been declared a public health emergency of international concern on January 30th 2020 by the World Health Organization (WHO) [1]. Figures from around the world show that approximately 7 to 11% of healthcare workers (HCWs) have been diagnosed with COVID-19 [2]. Political measures adopted to control the disease such as closure of childcare services, and disrupted socialization exacerbated existing inequities and posed additional challenges for HCW mothers (HCWM) with young children [3]. They had to balance work responsibilities such as providing patient care against their responsibilities to protect their own well-being from the effects of chronic stress and their children’s mental health [4]. HCWMs with young children raised their concerns about access to childcare, parenting, and household duties [5]. Inability to find caregivers and elderly parents for childcare support was a significant contributor to this inequity. While there is limited research on this specific topic, these challenges may have contributed to increased child behaviour problems particularly at a young age. In addition, as noted in the United Nations report on Turkey’s gender equality performance from 2000 to 2019, Türkiye still maintains a strong traditional division of labour. Although it is improving day by day, women still spend four times more time than men on unpaid domestic work (the double burden of housework and childcare), which is not sufficiently shared by family members, especially men, increasing women’s workload [6].

Children of HCWMs were disadvantaged relative to their peers due to deprivation of maternal care for an extended duration, the risk of their parents contracting the virus, the fear of losing their parents, and changes in family routines due to their parents’ work schedules [7]. Trauma faced at a young age may have long-term adverse developmental consequences across their lifespan [8].

Studies have shown that HCWs have experienced higher levels of stress, anxiety, and depression compared to the general population during the pandemic [9–11]. Notably, parental distress can be a significant contributor to child behavioural problems. Distressed parents tend to demonstrate emotional unavailability thus leading to the occurrence of child behaviour problems [9, 12]. On the other hand, parental distress has been shown to be associated with lack of social support. It has been shown that social support provided by family and friends can help reduce parental distress and have a positive effect on parenting behaviours. Due to social distancing measures these protective close relationships of families were cut off [10]. Social support plays an important role in a person’s mental health, and even more in disasters acts as a buffer against the negative effects of trauma-related events, minimizing the potential of developing negative consequences [11, 13]. Therefore, measuring the social support of HCWs would be of critical importance in planning interventions for this vulnerable population [7].

Overall, the pandemic has had a significant impact on the daily lives of children such as changes in daily routines, increased stress and anxiety, reduced physical activity, reduced peer interactions, increased screen time and changes in sleep environment which can contribute to sleep disturbances in young children [12, 14]. On the other hand, other studies have found that improved parent-child interactions due to home confinement could allow for better sleep [12, 14–16].

The study was planned based on the hypothesis that children of HCWMs experienced more socioemotional and behavioural (SEB) problems than children of mothers from other occupations during the COVID-19 pandemic. We aimed to explore SEB problems and sleep patterns in children of HCWMs; assess the psychological distress of mothers and their perceived social support during the COVID-19 pandemic, and compare them with age-matched peers and their mothers from other occupations.

## Materials and methods

A case-control study was applied and a snowball approach was used to enrol volunteered participants. All women having children aged between 6 and 36 months of age were invited to participate via websites, social media and e-mail between August 18th 2020 - October 17th 2020. All surveys were conducted in Türkiye. Personal identifying information was not collected or recorded.

An online survey was developed on the Google platform securing data collection by a confidential login system which can be filled out through smartphones, tablets or computers. The questionnaire took approximately 15 min to complete. It was pilot tested on 15 mothers and found acceptable. Due to pandemic restrictions, the study has been promoted via social media (such as Whatsapp, Telegram doctor and parent groups, Instagram, websites) and they are being asked to send links to volunteers in their area. Volunteers were sent an invitation email or message with a link to an anonymous online questionnaire, depending on their contact preferences. To access the questionnaire, respondents were asked to read the informed consent form and tick the mandatory box. Questionnaire responses were converted to excel spreadsheets.

**Fig. 1:**
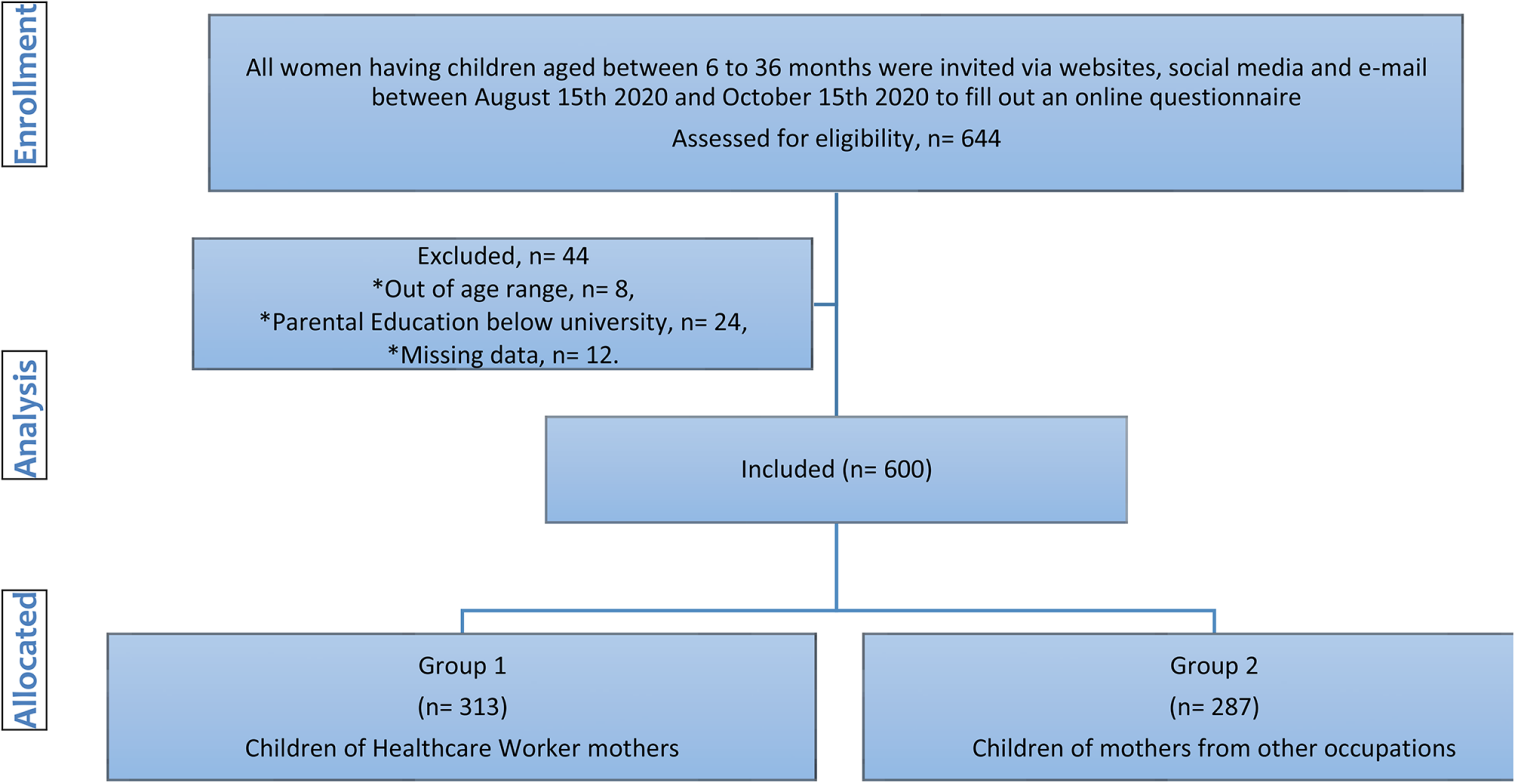
Flow chart of the study

Children aged 6–36 months and working mothers aged 20–45 years with at least some college/university education were eligible to participate in the study. Children whose parents didn’t approve the consent form or who had a known chronic health problem were excluded from the study. Children of HCWMs were grouped as Group 1 (HCWM group), and children of mothers from other occupations were grouped as Group 2 (non-HCWM group). Doctors, nurses, and paramedics are considered as HCWMs. For two groups, the difference between the groups was predicted to be statistically significant at the medium effect size (according to the percentage of SEB and sleep problems in that age group), and the total sample size was calculated as 600 for 95% power and 0.05 alpha significance level, taking into account dropouts and other possible problems (Fig. 1).

Brief Infant Sleep Questionnaire (BISQ) [17–20] and Brief Infant-Toddler Social and Emotional Assessment (BITSEA) questionnaires [21–23] were used to assess children’s sleep, and social-emotional development, respectively. Multidimensional Scale of Perceived Social Support (MSPSS) [24, 25], and Brief Symptom Inventory (BSI) [26, 27] questionnaires were used to evaluate maternal social support and psychological distress.

### Questionnaires

The BISQ functions as a tool designed to assess the early childhood sleep environment, covering aspects such as parental practices, daytime and night-time routines, and sleep issues, relying on responses from parents [17]. Through validation, BISQ demonstrated significant correlations with sleep measures obtained from actigraphy and sleep diaries. The Turkish translation of BISQ has been deemed acceptable, comprehensible, and reliable for evaluating sleep-related factors in infants [18]. In this study, maternal perception of sleep problems was categorized based on the expanded BISQ version. A parent-reported sleep problem was defined as moderate or severe while no problem was designated for those with no problem or a very small problem, as indicated in previous studies [17, 19]. Parents were asked a single question to assess the pandemic’s impact on their children’s sleep. The question of whether there was a change in their children’s sleep during the pandemic period and if the answer is “yes”, what has changed was added to the beginning of the BISQ survey. If the child woke up > 3 times/night and woke up after sleep onset (WASO) for > 1 h or spent < 1 SD of the study population’s sleep duration (10 h), the child was considered a poor sleeper [17, 20].

The BITSEA is a tool for screening social, emotional and behavioural development in early childhood which consists of 42 items [21]. The BITSEA encompasses two scales: the Problem scale (BITSEAp) with 31 items and the Competence scale (BITSEAc) with 11 items. Each item utilizes a response format with three choices: “not true/rarely” (0), “sometimes true/sometimes” (1), and “very true/often (2).” Problematic behavior is addressed in the externalizing (6 items, e.g., impulsivity, defiance, peer aggression), internalizing (8 items, e.g., fearfulness, worry, anxiety, sadness), and dysregulation (8 items, e.g., sleep and eating problems, negative emotionality, sensory sensitivities) domains [22]. Higher total scores on BITSEAp indicate a greater level of behavioral and emotional problems, while lower total scores on BITSEAc indicate a lower level of competence. The Turkish version of BITSEA has demonstrated reliability, validity, and simplicity, making it an effective instrument for screening social, emotional, and behavioral problems in toddlers [23]. Identified cut-offs of BITSEAp in gender groups for subclinical scores were ≥ 18 for males, and ≥ 21 for females. It was ≥ 24 for clinical scores in both genders [21, 23].

The MSPSS is a concise self-administered tool comprising 12 items, divided into three subscales [24]. Respondents rate each item on a seven-point Likert scale, ranging from 1 (very strongly disagree) to 7 (very strongly agree). A higher score on the Likert scale indicates a greater perception of social support. The total score, ranging from 12 to 84, can be calculated by adding the items within each subscale and then dividing by 4. The instrument assesses the level of social support and identifies its sources from family, friends, or significant others. Elevated scores are indicative of higher levels of perceived social support. The Turkish adaptation of MSPSS has demonstrated reliability, validity, and ease of application, establishing it as an effective tool for screening perceived social support [25].

The BSI comprises a self-report symptom inventory with 53 items, encompassing nine symptom dimensions: Somatization, Obsession-Compulsion, Interpersonal Sensitivity, Depression, Anxiety, Hostility, Phobic Anxiety, Paranoid Ideation, and Psychoticism; along with three global distress indices: Global Severity Index, Positive Symptom Distress Index, and Positive Symptom Total [26]. This instrument facilitates the evaluation of psychological distress and psychiatric disorders. Respondents rate items on a five-point Likert scale, ranging from 0 (not at all) to 4 (extremely), indicating the degree of distress experienced in the past week. Scores are generated for nine primary symptom dimensions and three global distress indices. The Turkish adaptation of BSI has demonstrated reliability, validity, and simplicity, making it a valuable tool for assessing maternal psychological distress, including depression, anxiety, hostility, negative self-perception, somatization, and global distress indices [27].

### Statistical analyses

IBM SPSS Statistics software (version 28.0, IBM Inc., United States) was employed for all analyses. Descriptive statistics, including mean and standard deviations (SD) for normally distributed continuous variables, and median and quartiles for non-normally distributed data, were presented. Normal distribution conformity was assessed using Kolmogorov-Smirnov and Shapiro-Wilk tests. Categorical variables were summarized using frequencies and percentages. Differences in family characteristics, sleep parameters, BITSEA, BSI, and MSPSS scores were examined using the chi-square and Mann-Whitney U test, as appropriate. Given the non-normal distribution of all parameters, Spearman’s Rho Correlation analysis test was utilized to calculate correlation coefficients and assess significance.

Adjusted odds ratios (ORs) and 95% confidence intervals (CIs) were reported. To compare study group data with nationally representative data, a one-sample T-test and chi-square test were applied for sleep parameters, and the chi-square test was used for SEB problem frequency. In multivariate analysis, factors identified in univariate analysis, without collinearity, were entered into logistic regression to determine independent predictors of children’s behavioral and emotional problems. The Hosmer–Lemeshow test assessed the goodness-of-fit of the model. A 5% type-I error level was set for determining statistical significance.

## Results

In total, 600 questionnaires were analysed, 313 children were included in the HCWM group and 287 were included in the non-HCWM group (Fig. 1). There were no statistically significant differences in maternal and paternal occupation between the groups. Although the mothers who had a college/university education and above were included in the study, there was a statistically significant difference between the groups according to the level of parental education. Although the median age of children was significantly higher in the HCWM group (*p* = 0.005), age groups (< 24 months vs. 24–36 months) and gender distribution was similar in both groups (*p* = 0.133, *p* = 0.416).The sociodemographic and parental data of groups are summarized in Table 1.

**Table 1:** Clinical characteristics, sociodemographic parental data and BITSEA, BSI and MSPSS scores of study participants

	Group 1 (*n* = 313)	Group 2 (*n* = 287)	*p*
**Maternal age (years), median (IQR)**	33.0 (5)	30.0 (5)	0.792^*^
**Maternal Education, n (%)**			
College/University	117 (37.4)	203 (70.7)	**< 0.001** ^**^
Graduate (MS, MD, PhD)	196 (62.6)	84 (29.3)	
**Maternal occupation n (%)** ^**¥**^			
1. Professional	176 (56.2)	168 (58.5)	0.626^**^
2. Non-manuel skilled	116 (37.1)	93 (32.4)	
3. Manuel Skilled	11 (3.5)	11 (3.8)	
4. Partly skilled	7 (2.2)	11 (3.8)	
5. Unskilled	0 (0)	1 (0.3)	
6. Housewife	3 (1)	3 (1)	
**Employed in a Pandemic Hospital, n (%)**	134 (42.8)	-	**-**
**Employment status during the pandemic**, n (%)			
On leave,	62 (19.8)	137 (47.7)	**< 0.001** ^**^
Working	251 (80.2)	150 (52.3)	**1.53 (1.35–1.73)** ^**#**^
**Paternal age (years), median (IQR)**	34.0 (7)	31.0 (4)	0.289^*^
**Paternal Education, n (%)**			
High school	25 (8)	22(7.6)	**< 0.001** ^**^
College/University	137 (43.8)	176 (61.3)	
Graduate (MS, MD, PhD)	151 (48.2)	89 (31.1)	
**Family size, median (IQR)**	3 (1)	3 (0)	**< 0.001** ^*^
< 5 members	262 (83.7)	270 (94.1)	**< 0.001** ^**^
5 and above	51 (16.3)	17 (5.9)	
**Family type, Nuclear, n (%)**	186 (62.6)	245 (80.9)	**< 0.010** ^**^
**Child number, Only child, n (%)**	217 (69.3)	220 (76.6)	**0.050** ^**^
**Child age (years), median (IQR)**	2.3 (1.0)	1.6 (1.2)	**0.005** ^*^
< 24 months, n (%)	163 (52.1)	167 (58.2)	0.133^**^
24–36 months, n (%)	150 (47.9)	120 (41.8)	
**Male sex, n (%)**	153 (48.9)	146 (50.9)	0.416^**^
**Breastfeeding status currently** **(for children < 24 months), n (%)**	108 (66.3)	120 (71.4)	0.310^**^
**BITSEA, total score, median (IQR)** ^*^	33.0 (10.2)	26.0 (13.0)	**0.031** ^*^
**BITSEAp**	15.0 (7.5)	13.0 (8.0)	0.224^*^
BITSEAp externalizing	5.0 (4)	4 (3)	**0.031** ^*^
BITSEAp internalizing	4 (3)	4 (4)	**0.003** ^*^
BITSEAp dysregulation	2 (3)	2 (2)	**0.016** ^*^
**BITSEAc**	17.0 (4.0)	16.0 (9.0)	**0.030** ^*^
**BITSEAp above clinic cut-off**	49 (15.7)	32 (11.1)	0.107^**^
**BITSEAp above subclinical cut-off**	96 (30.7)	67 (23.3)	**0.044** ^**^
**BSI, median (IQR)**			
Anxiety	14.0 (19.0)	11.0 (25.0)	0.321^*^
Depression	14.0 (20.0)	13 (24.0)	0.480^*^
Hostility	6.0 (9.0)	9.0 (10.0)	0.524^*^
Somatization	5.5 (10.0)	6.0 (10.0)	0.077^*^
Negative self	9.0 (18.0)	11.0 (18.0)	0.618^*^
Global index	0.9 (1.2)	0.9 (1.4)	0.511^*^
**MSPSS, total score**			
Family, median (IQR)^*^	6.2 (2.0)	6.0 (1.5)	0.857^*^
Friends, mean (SD)	5.2 (2.5)	5.5 (2.2)	0.142^***^
Significant others, mean (SD)	4.5 (3.0)	5.7 (4.0)	0.384^***^

*Mann-Whitney U test, ** Chi-square test, ^***^T-test, ^#^(OR, 95% CI), ¥ according to ILO classification

SD: standard deviation, IQR: interquartile range, BITSEA: Brief Infant–Toddler Social and Emotional Assessment, BITSEAp: Brief Infant–Toddler Social and Emotional Assessment Problem Scale, BITSEAc: Brief Infant–Toddler Social and Emotional Assessment Competence Scale, BSI: Brief Symptom Inventory, MSPSS: Multidimensional Scale of Perceived Social Support

Changes in the daily routines and family members’ COVID-19 exposure status were presented in Table 2. A significantly higher proportion of mothers in the HCWM group were separated from their children during the pandemic (HCWM vs. non-HCWM: 27.8% vs. 10.8%). Moreover, the duration of separation was significantly longer (20 vs. 3 days, *p* = 0.002) in the HCWM group. Childcare support by nannies and grandparents and changes in the child’s structured outdoor activities during the pandemic period were more likely observed in the HCWM group (*p* < 0.05).

**Table 2:** Changes in daily routines during the pandemic of study participants

	Group 1 (*n* = 313)	Group 2 (*n* = 287)	*p / OR (95% CI)*
**Social stressors, n (%)**	63 (20.1)	64 (22.2)	0.549^*^
Income loss	6 (1.9)	11 (3.8)	
Marital struggle	55 (17.6)	47 (16.4)	
Job lost	2 (0.6)	6 (2.1)	
**Separated from her child, n (%)**	87 (27.8)	31 (10.8)	**< 0.001** ^*^ 2.57 (1.76–3.75)
**Duration of separation (days) from the child**^&^, **median (IQR)**	20 (56)	3 (14)	**0.002** ^**^
**Change in childcare during the pandemic, n (%)**	166 (53)	135 (47)	0.165^*^
Could not help from their grandparents	34 (10.9)	50 (17.4)	
The nanny could not come	29 (9.2)	31 (10.8)	
I left my child with others for care	37 (11.8)	18 (2.8)	
Other	66 (21.1)	46 (16.)	
**Childcare support, n (%)**	188 (60.1)	148 (51.6)	**0.04** ^*^ **1.16 (1.00-1.34)**
None	125(39.9)	139 (48.4)	
Live-in nanny	20 (6.4)	6 (2.1)	
Daytime nanny	48 (15.3)	34 (11.8)	
Grandparent	73 (23.4)	55 (19.1)	
Father	47 (15)	53 (18.5)	
**Any structured activity, n (%)** **None**	222 (70.9)	230 (80.1)	**0.01** ^*^ **0.88 (0.80–0.97)**
**Change in structured activity the during pandemic, n (%)**	91 (29.1)	57 (19.9)	**0.01** ^*^ **1.46 (1.09–1.95)**
No Day-care	42 (13.4)	15 (5.2)	
No outdoor activity	34 (10.9)	35 (12.2)	
Other	16 (5.3)	2 (0.7)	
**Exposure to COVID-19 in the family, n (%)**	22 (7)	19 (6.6)	0.873^*^
Isolated at home	16 (5.1)	14 (4.9)	
Hospitalized	6 (1.9)	5 (1.7)	

* Chi-square test, ** Mann-Whitney u test, & the separated ones were included in the analysis

### BITSEA

**Fig. 2:**
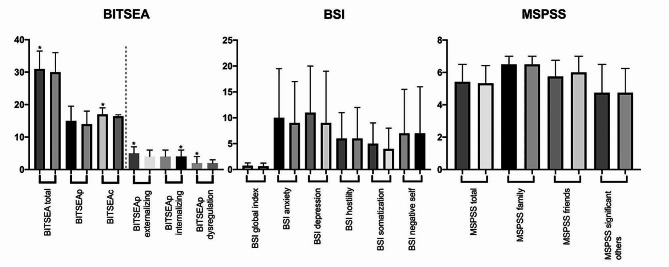
BITSEA, BSI and MSPSS scores for each subscale [* *p* < 0.05 (Mann-Whitney u test); For each scale first column indicates Group 1 and second column indicates Group 2; BITSEA: brief infant–toddler social and emotional assessment, BITSEAp: The brief infant–toddler social and emotional assessment problem scale, BITSEAc: brief infant–toddler social and emotional assessment competence scale, BSI: Brief Symptom Inventory, MSPSS: Multidimensional Scale of Perceived Social Support]

**Fig. 3:**
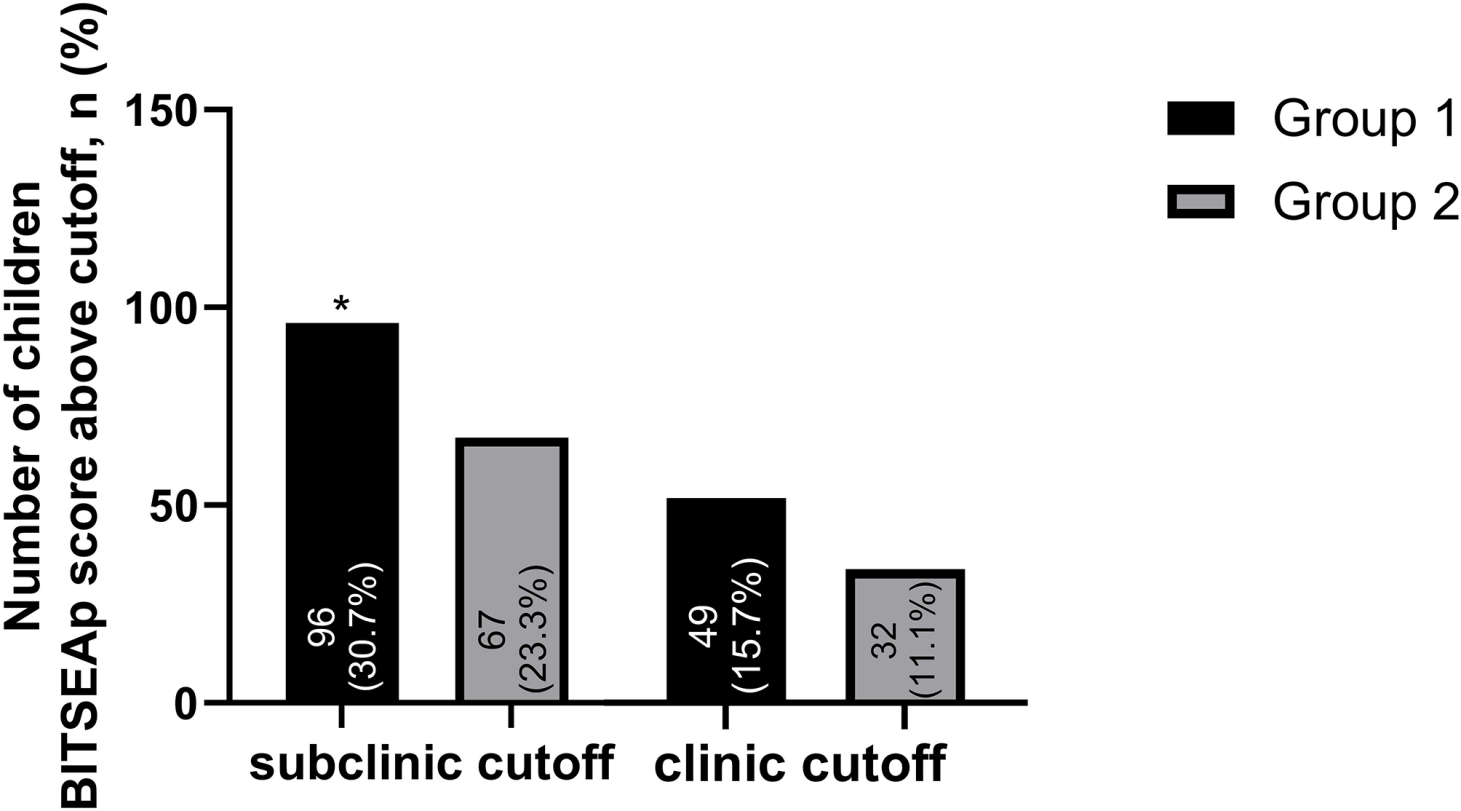
Distribution of BITSEAp scores above the cut-off [* *p* < 0.05 at Chi-square test, BITSEAp: The brief infant–toddler social and emotional assessment problem scale, identified cut-offs of BITSEA/P in gender groups for subclinical scores were ≥ 18 for males and ≥ 21 for females. It was ≥ 24 for clinical scores in both genders]

Total and subscale scores of the BITSEA, BSI and MSPSS are presented in Fig. 2; Table 1. Although there were no significant differences in children’s BITSEAp median scores between groups, the externalizing and dysregulation scores were significantly higher in the HCWM group (*p* = 0.031, *p* = 0.016). There were no significant differences in the BITSEAp and BITSEAc scores between boys and girls (boys vs. girls median BITSEAp 15 vs. 14, *p* = 0.461; median BITSEAc 17 vs. 17, *p* = 0.093). The median BITSEAc and total score were significantly higher in the HCWM group (*p* = 0.030, *p* = 0.031). Although children above BITSEAp subclinical cut-off were found significantly higher in the HCWM group (30.7% vs. 23.3%, *p* = 0.044), there was no significant difference between clinical cut-off points. There were no significant differences in mothers’ BSI and MSPSS total and subscale median scores between the groups (Fig. 3; Table 1).

While other BSI subscales and BITSEAp had a mild positive correlation, BSI global index and BITSEAp had a positive moderate correlation (*r* = 0.412, *p* < 0.001). All BSI subscales (anxiety, depression, hostility, somatization, negative-self and global index) and BITSEAp subscales (externalizing, internalizing and dysregulation) had a mild positive correlation (*p* < 0.001). Additionally, the MSPSS total and BSI global index had a negative moderate correlation (*r*=-0.430, *p* < 0.001). It was shown that almost all MSPSS and BSI subscales had negative mild to moderate correlations. Bivariate correlation of BITSEA, BSI, MSPSS subscales, sleep parameters and associated sociodemographic variables are summarized in Supplemental File Table 1.

Although there were no significant differences between study groups, it was shown that the percentage of SEB problems in HCWM’s children was significantly higher than national representative data (13.5% vs. 11.9%, with a chi-square test *p* < 0.001) [28].

In the logistic analysis, male gender (*p* < 0.001, RR: 3.68, 95% CI: 1.99–6.80), each point increases in BSI global index scores (*p* < 0.001, RR: 2.56, 95% CI: 1.61–4.09), and having HCWM (*p* = 0.018, RR: 2.27, 95% CI: 1.15–4.48) were found as the risk factors for BITSEAp scores above the subclinical cut-off. Besides, each point increases in BSI global index scores (*p* < 0.001, RR: 3.34, 95% CI: 1.91–5.82) was found as a risk factor for clinically significant SEB problems (Table 3).

**Table 3:** Associations of variables on BITSEAp scores above sub-clinic and clinic cut-off

	BITSEAp above the sub-clinic cut-off			BITSEAp above the clinic cut-off		
**Variables**	P value	RR	95% CI	P value	RR	95% CI
Gender (male)	**< 0.001**	**3.68**	**1.99–6.80**	0.248	1.58	0.73–3.42
Age (year)	0.688	0.91	0.58–1.41	0.286	0.74	0.42–1.29
Child number	0.998	0.99	0.54–1.86	0.321	1.49	0.68–3.25
Maternal Education	0.498	0.80	0.43–1.51	0.916	0.96	0.43–2.12
Change in structured activity during a pandemic	0.065	0.51	0.25–1.04	0.899	1.05	0.44–2.57
Change in childcare support during a pandemic	0.845	1.07	0.56–2.01	0.525	0.76	0.33–1.75
Change in children’s sleep during a pandemic	0.673	1.14	0.62–2.13	0.113	1.87	0.86–4.07
BSI global index	**< 0.001**	**2.56**	**1.61–4.09**	**< 0.001**	**3.34**	**1.91–5.82**
MSPSS total	0.906	1.02	0.70–1.29	0.588	0.92	0.69–1.24
Separated from her child	0.731	0.99	0.98–1.03	0.922	1.01	0.98–1.02
Having HCW mother	0.018	2.27	1.15–4.48	0.203	1.77	0.73–4.30
Constant	0.012	-2.73		0.013	-3.34	

Factors that were statistically significant in predicting BITSEAp in bivariate analysis were entered in the multiple logistic regression model

Abbreviations: BSI: Brief symptom inventory, MSPSS: Multidimensional Scale of Perceived Social Support, HCW: Healthcare worker, RR: Relative Risk Ratio, CI: Confidence Interval

### Sleep

Table 4 provides a summary of maternal perceptions regarding sleep problems and sleep variables based on BISQ. It reveals that both total and night-time sleep durations were significantly shorter in the HCWM group. Furthermore, the HCWM group demonstrated a significantly higher prevalence of perceiving their child’s sleep as a moderate to severe problem.

**Table 4:** Maternal perception of sleep problems and sleep variables according to BISQ

	Group 1 (313)	Group 2 (287)	*p / OR(95%CI)*
Changes in children’s sleep habits during the pandemic, n(%)			
**Changes in sleep after the pandemic**	131 (41.5)	36 (30)	**0.004** ^*^ **1.38 (1.11–1.72)**
**Sleep later**	24 (7.7)	25 (8.7)	0.657^*^
**Increased night wakening**	73 (23.3)	37 (12.9)	**0.001** ^*^ **1.80 (1.26–2.59)**
**Reduced night-time sleep**	29 (9.3)	18 (6.3)	0.223^*^
**Increased sleep onset latency**	26 (8.3)	21 (7.3)	0.761^*^
**Increased early morning wakening**	10 (3.2)	5 (1.7)	0.303^*^
**Nap problems**	6 (1.9)	9 (3.1)	0.435^*^
**Sleep better**	3 (1)	6 (2.1)	0.323^*^
**Maternal perception of sleep problem, n (%) and BISQ sleep variables**			
**Perception of child’s sleep, n(%)**			**0.010** ^*^ **1.51 (1.10–2.08)**
** Moderate to severe problem**	81 (25.9)	49 (17.1)	
** Not a problem/A small problem**	232 (74.1)	238 (82.9)	
**Bedtime hour (clock time), median (IQR)**	21:59 (2:00)	21:30 (1:45)	0.175^**^
**Wake-up hour (clock time), median (IQR)**	06:59 (1:30)	07:30 (1:00)	**0.004** ^**^
**Night awakening’s, median (IQR)**	2.0 (2.0)	2.0 (2.0)	0.354^**^
**The longest stretch of night-time sleep (minute), median (IQR)**	240.0 (240.0)	300.0 (240.0)	0.974^**^
**WASO (minute), median (IQR)**	30.0 (64.0)	30.0 (45.0)	0.130^**^
**Night-time sleep duration (hour), median (IQR)**	9.0 (2.0)	10.0 (2.0)	**0.003** ^**^
**Daytime sleep duration (minute), median (IQR)**	120.0 (34.0)	120.0 (60.0)	0.095^**^
**Total sleep duration (hour), median (IQR)**	11.0 (2.5)	12.0 (1.2)	**0.003** ^**^
**Poor Sleeper, n (%)**	80 (25.6)	57 (19.9)	0.097**

*Chi-square test, **Mann-Whitney u test, BISQ: Brief Infant Sleep Questionnaire, WASO: wake after sleep onset, IQR: interquartile range.

Changes in sleep patterns and sleep disturbances in children pre- versus post-pandemic were analysed. Changes in sleep pattern were 1.38 times and night wakening’s were 1.8 times higher as compared to the pre-pandemic period in the HCWM group (HCWM vs. non-HCWM group: 41.5% vs. 30% *p* = 0.004, OR: 1.38; 23.3% vs. 12.9% *p* = 0.001, OR: 1.80). HCWMs were more likely to perceive their children’s sleep as a moderate to severe problem compared to the non-HCWM group (25.9% vs. 17.1%, *p* = 0.01, OR: 1.51). Wake-up time was significantly earlier, and night-time and total sleep duration were significantly shorter in the HCWM group (*p* = 0.004, *p* = 0.003, *p* = 0.003). Overall, 22.8% of the study population were classified as poor sleepers and there was no significant difference between the groups (*p* = 0.097).

Compared with the recent study that represents Türkiye’s nationwide sleep characteristics of the same age group, whole study groups differed significantly from the normative distribution in almost all sleep parameters (Night waking’s increased by 2.63 vs. 2.3, *p* < 0.001; decreased night-time sleep duration 9.3 vs. 9.6 h, *p* < 0.001; shorter total sleep duration 11.2 vs. 11.5 h, *p* = 0.002; increased WASO 59.1 vs. 48.1 min, *p* = 0.01; decreasing nap number 1.4 vs. 1.6, *p* < 0.001) [29].

## Discussion

There is limited research available on whether children of HCWMs exhibit more SEB problems compared to their peers during the pandemic. Overall, the current study suggests HCWMs have experienced occupational inequality, and young children of HCWMs were at increased risk for externalizing and dysregulation problems during the pandemic. Our findings showed a significantly higher proportion of HCWMs were separated from their children for longer periods. Disrupted childcare support and changes in daily routines have disproportionally impacted HCWMs. Children of HCWMs had higher scores on the externalizing and dysregulation domains of SEB problems than children of non-HCWMs. On the other hand, psychological distress and perceived social support were not different in HCWMs compared to non-HCWMs. Moreover, competence scores were significantly higher in children of HCWMs, suggesting young children of HCWMs may have experienced positive outcomes as well. Maternal psychological distress was found as a potentially modifiable factor affecting the child’s SEB problems. Our findings also showed that these children of HCWMs had higher parent-reported sleep problems, shorter total and night-time sleep, and earlier wake time compared to their peers of non-HCW parents. Moreover, they had an increase in their night wakening and changes in sleep patterns compared to the pre-pandemic period.

Parent-reported SEB problems in infancy and throughout toddlerhood have been reported between 5 and 24% in different studies before the pandemic period [30, 31]. Children of HCWs faced unique stressors, including the risk of their parents contracting the virus, changes in family routines due to their parents’ work schedules, and separation from their parents due to long working hours or quarantine measures. While there is limited research suggesting that children of HCWs may be at increased risk of experiencing SEB problems during the pandemic, the available evidence shows that these stressors can contribute to SEB problems in young children. It is known that externalizing problems are mainly encountered in early childhood because they have limited abilities to communicate about their emotions and use externalizing behaviours to express their emotions [32]. The findings of our study showed young children of HCWMs faced additional stressors such as separation from their parents and disruption of their daily routines due to their parent’s occupations. We also found that children of HCWMs had higher externalizing and dysregulation behaviours compared to their peers. Overall, clinically significant SEB problem was found in 13.5% of the children. The study from Türkiye has shown that parent-reported clinically significant SEB problem was observed in 11.9% of Turkish children aged between 2 and 3 years [28]. In the validation and reliability study of the BITSEA Turkish version, clinically significant SEB problem was found at 13.1% for males and 17.6% for females [23]. In this current study, although there were no significant differences between groups, it was shown that the percentage of SEB problems in HCWM’s children was significantly higher than the nationally representative data [28]. Further, the number of children above BITSEAp subclinical cut-off was found significantly higher in the HCWM group suggesting subclinical difficulties at younger ages may also increase the risk for later psychopathology [32, 33].

Research findings have been mixed and some studies have suggested that children of HCWs may experience positive outcomes as well [34]. Similarly, the current study found that competence scores were significantly higher in children of HCWMs, suggesting young children of HCWMs may have experienced positive outcomes as well. It is important to note that the impact may vary depending on the type of healthcare work their parents are engaged in, the family’s overall social and economic circumstances, and the child’s level of resilience and coping strategies. The current study has not shown a significant difference in perceived social support between HCWMs and non-HCWMs. Social support can take many forms including emotional support, and practical support. It can be particularly important for parents who are experiencing additional challenges. Further research is needed to fully understand the issue.

SEB problems have been linked with high maternal depression and parenting stress [33]. Stress and anxiety levels of parents are associated with children’s SEB [35] and psychological development [36]. During stressful events, children’s emotional adjustment develops and changes mostly depending on the emotional state of their parents [37]. It has been suggested that mothers with depressive symptoms have difficulty responding appropriately in interactions with their children, show less sensitivity to cues from their children, participates less in positive interactions with their children, and cannot use the right strategies to manage their children’s behaviour [38]. Higher total BSI scores of the primary caregiver and being separated from the mother for more than a month were considered clinically significant risk factors for SEB problems in children [39]. Similarly, the study findings showed that higher maternal psychological distress levels were significantly associated with clinically significant SEB problems.

The COVID-19 pandemic has had a significant impact on the daily lives of young children which can contribute to sleep disturbances. In a recent study, it was shown that altered daily routines due to COVID-19 confinement, caused worsened sleep quality in children aged 0–36 months and the caregiver’s stress level was identified as a significant risk factor for this impairment [40]. Similarly, when the parents were asked about the reason for the altered sleep habits, they reported their children slept later than usual, sleep routines were disrupted, did not nap during the day, and had changes in feeding routines [41]. Our study showed that young children of HCWMs faced increased night wakening, earlier wake-up times and shorter night-time and total sleep times than peers. Compared with the recent study that represents Türkiye’s nationwide sleep characteristics of the same age group, almost all the sleep parameters were significantly worse than the normative distribution [29]. Additionally, HCWMs reported that their children’s sleep patterns changed compared to the pre-pandemic period. Sleep problems were found to be associated with SEB problems in the first 3 years of life [42]. Changes in daily routines, and sleep patterns might have contributed to SEB problems in young children who are particularly at a critical stage of social and emotional development. Parents need to establish consistent routines and support healthy sleep habits for young children to address these challenges.

It is important to note that this study only included young children living in Türkiye, and may not be representative of all children of HCWMs. The case-control design of our study did not let us provide information about the causality of SEB and sleep problems. Due to the snowball sampling approach, the generalizability of these findings is limited. To avoid this limitation, we calculated and reached the minimum sampling size to represent our population. The measures used for this study were self-reported. Reliable and valid questionnaires for our country had been used to overcome this limitation. Because the number of children above the clinical cut-off is limited, the significant difference shown in the subclinical cut-off may have not been demonstrated at the clinical cut-off. More research is needed to fully understand the impact of the pandemic on the SEB problems and the well-being of children of HCWMs. The differences between the groups in terms of parental education and family type were notable, although having at least a university/college education was an inclusion criterion. Our aim was to assess mothers’ perceptions but the lack of data on fathers is also a limitation of this study.

The findings of this study suggest that young children of HCWMs were disproportionally affected due to their mothers’ occupations during the COVID-19 pandemic. Addressing psychological distress in HCWMs, improving social support and implementing structured daily routines may serve as potential protective factors for behavioural functioning in these children and thus reduce occupation-related inequalities during the crisis. There is a need for further research into occupational inequality and its potential impact on social and emotional factors particularly for children and parents.

### Electronic supplementary material

Below is the link to the electronic supplementary material.

**Supplementary Material 1**: Bivariate correlation of variables

## Data Availability

The datasets generated during and/or analysed during the current study are available from the corresponding author on reasonable request.
