# Supplementary material for: Social-emotional and behavioural problems in young children of healthcare worker mothers during the COVID-19 outbreak: a case-control study
Source: BMC Psychiatry. 2024 May 30;24:407. doi: 10.1186/s12888-024-05801-4 (PMC11137945; doi:10.1186/s12888-024-05801-4)
Supplement: Supplementary file 1 — Supplementary Material 1: Bivariate correlation of variables [file 12888_2024_5801_MOESM1_ESM.docx]

**Supplementary File Table 1.** Bivariate correlation of variables

|  | | | 1 | 2 | 3 | 4 | | | 5 | 6 | 7 | 8 | 9 | 10 | 11 | 12 | 13 | 14 | 15 | 16 | 17 | 18 | 19 | 20 | 21 | 22 | 23 | 24 | 25 | 26 | 27 | 28 |  |
| --- | --- | --- | --- | --- | --- | --- | --- | --- | --- | --- | --- | --- | --- | --- | --- | --- | --- | --- | --- | --- | --- | --- | --- | --- | --- | --- | --- | --- | --- | --- | --- | --- | --- |
| 1.child age | | | 1,000 |  |  |  | | |  |  |  |  |  |  |  |  |  |  |  |  |  |  |  |  |  |  |  |  |  |  |  |  |  |
| 2.gestational age | | | 0,052 | 1,000 |  |  | | |  |  |  |  |  |  |  |  |  |  |  |  |  |  |  |  |  |  |  |  |  |  |  |  |  |
| 3.child number | | | 0,121^**^ | -0,119^**^ | 1,000 |  | | |  |  |  |  |  |  |  |  |  |  |  |  |  |  |  |  |  |  |  |  |  |  |  |  |  |
| 4.family size | | | 0,126^**^ | -0,130^**^ | **0,712^**^** | 1,000 | | |  |  |  |  |  |  |  |  |  |  |  |  |  |  |  |  |  |  |  |  |  |  |  |  |  |
| 5.maternal age | | | 0,223^**^ | -0,098^*^ | **0,384^**^** | 0,282^**^ | | | 1,000 |  |  |  |  |  |  |  |  |  |  |  |  |  |  |  |  |  |  |  |  |  |  |  |  |
| 6.paternal age | | | 0,193^**^ | -0,087^*^ | **0,365^**^** | 0,278^**^ | | | **0,749^**^** | 1,000 |  |  |  |  |  |  |  |  |  |  |  |  |  |  |  |  |  |  |  |  |  |  |  |
| 7.separated day | | | 0,172 | -0,037 | 0,160 | 0,073 | | | 0,071 | 0,086 | 1,000 |  |  |  |  |  |  |  |  |  |  |  |  |  |  |  |  |  |  |  |  |  |  |
| 8.BITSEAtotal | | | 0,191^**^ | -0,029 | -0,016 | 0,009 | | | -0,039 | -0,045 | 0,057 | 1,000 |  |  |  |  |  |  |  |  |  |  |  |  |  |  |  |  |  |  |  |  |  |
| 9.BITSEAp | | | 0,017 | -0,053 | -0,038 | -0,024 | | | -0,114^**^ | -0,089^*^ | 0,025 | **0,873^**^** | 1,000 |  |  |  |  |  |  |  |  |  |  |  |  |  |  |  |  |  |  |  |  |
| 10.BITSEAc | | | **0,418^**^** | 0,082 | 0,043 | 0,067 | | | 0,128^**^ | 0,092^*^ | 0,065 | **0,323^**^** | -0,092^*^ | 1,000 |  |  |  |  |  |  |  |  |  |  |  |  |  |  |  |  |  |  |  |
| 11.BITSEAp externalizing | | | -0.043 | -0.077 | -0.042 | -0.001 | | | -0.063 | -0.044 | -0.055 | **0.688^**^** | **0.764^**^** | -0.064 | 1.000 |  |  |  |  |  |  |  |  |  |  |  |  |  |  |  |  |  |  |
| 12.BITSEAp internalizing | | | 0.031 | -0.075 | 0.018 | 0.032 | | | -0.029 | -0.025 | 0.058 | **0.753^**^** | **0.805^**^** | -0.051 | **0.608^**^** | 1.000 |  |  |  |  |  |  |  |  |  |  |  |  |  |  |  |  |  |
| 13. BITSEAp dysregulation | | | 0.102^*^ | -0.050 | -0.044 | -0.047 | | | -0.076 | -0.060 | 0.089 | **0.669^**^** | 0.772^**^ | -0.112^**^ | **0.547^**^** | **0.580^**^** | 1.000 |  |  |  |  |  |  |  |  |  |  |  |  |  |  |  | ś |
| 14.BSI anxiety | | | -0,021 | -0,105^*^ | -0,035 | -0,023 | | | -0,003 | -0,033 | 0,064 | **0,311^**^** | **0,344^**^** | -0,048 | **0.311^**^** | **0.329^**^** | **0.305^**^** | 1,000 |  |  |  |  |  |  |  |  |  |  |  |  |  |  |  |
| 15.BSI depression | | | 0,017 | -0,117^**^ | -0,057 | -0,061 | | | -0,017 | -0,037 | 0,048 | **0,347^**^** | **0,387^**^** | -0,050 | **0.328^**^** | **0.356^**^** | **0.338^**^** | **0,849^**^** | 1,000 |  |  |  |  |  |  |  |  |  |  |  |  |  |  |
| 16.BSI hostility | | | -0,067 | -0,048 | -0,066 | -0,080 | | | -0,126^**^ | -0,111^**^ | 0,017 | **0,304^**^** | **0,372^**^** | -0,128^**^ | **0.305^**^** | **0.307^**^** | 0.298^**^ | **0,729^**^** | **0,766^**^** | 1,000 |  |  |  |  |  |  |  |  |  |  |  |  |  |
| 17.BSI somatization | | | -0,031 | -0,082 | -0,013 | -0,035 | | | -0,073 | -0,074 | 0,057 | **0,334^**^** | **0,388^**^** | -0,102^*^ | **0.303^**^** | **0.361^**^** | **0.325^**^** | **0,723^**^** | **0,737^**^** | **0,707^**^** | 1,000 |  |  |  |  |  |  |  |  |  |  |  |  |
| 18.BSI negative self | | | -0,042 | -0,086^*^ | -0,028 | -0,016 | | | -0,060 | -0,073 | 0,028 | **0,322^**^** | **0,386^**^** | -0,102^*^ | **0.336^**^** | **0.348^**^** | **0.311^**^** | **0,838^**^** | **0,867^**^** | **0,773^**^** | **0,718^**^** | 1,000 |  |  |  |  |  |  |  |  |  |  |  |
| 19.BSI global index | | | -0,031 | -0,100^*^ | -0,050 | -0,049 | | | -0,059 | -0,073 | 0,044 | **0,358^**^** | **0,412^**^** | -0,085^*^ | **0.355^**^** | **0.376^**^** | **0.347^**^** | **0,926^**^** | **0,942^**^** | **0,861^**^** | **0,819^**^** | **0,934^**^** | 1,000 |  |  |  |  |  |  |  |  |  |  |
| 20.MSPSS total | | | -0,059 | -0,001 | -0,025 | -0,008 | | | -0,009 | -0,009 | -0,017 | -0,192^**^ | -0,239^**^ | 0,107^**^ | -0.227^**^ | -0.231^**^ | -0.237^**^ | **-0,366^**^** | **-0,459^**^** | **-0,363^**^** | -0,293^**^ | **-0,428^**^** | **-0,430^**^** | 1,000 |  |  |  |  |  |  |  |  |  |
| 21.MSPSS family | | | -0,067 | 0,009 | -0,057 | -0,012 | | | -0,080 | -0,031 | 0,110 | -0,194^**^ | -0,238^**^ | 0,104^*^ | -0.230^**^ | -0.240^**^ | -0.212^**^ | **-0,368^**^** | **-0,473^**^** | **-0,377^**^** | **-0,303^**^** | **-0,435^**^** | **-0,441^**^** | **0,724^**^** | 1,000 |  |  |  |  |  |  |  |  |
| 22.MSPSS friends | | | -0,035 | 0,056 | -0,029 | -0,025 | | | 0,034 | 0,045 | -0,033 | -0,140^**^ | -0,201^**^ | 0,148^**^ | -0.168^**^ | -.0196^**^ | -0.225^**^ | -0,277^**^ | **-0,341^**^** | -0,294^**^ | -0,248^**^ | **-0,323^**^** | **-0,332^**^** | **0,793^**^** | **0,530^**^** | 1,000 |  |  |  |  |  |  |  |
| 23.MSPSS significant other | | | -0,073 | -0,030 | -0,012 | -0,005 | | | 0,005 | -0,023 | -0,112 | -0,166^**^ | -0,181^**^ | 0,032 | -0.199^**^ | -0.176^**^ | -0.184^**^ | **-0,306^**^** | **-0,371^**^** | -0,277^**^ | -0,216^**^ | **-0,344^**^** | **-0,344^**^** | **0,900^**^** | **0,499^**^** | **0,545^**^** | 1,000 |  |  |  |  |  |  |
| 24. Wake-up hour | | | 0,232^**^ | 0,058 | 0,063 | 0,051 | | | -0,014 | -0,026 | -0,005 | 0,014 | -0,075 | 0,143^**^ | -0,121^**^ | -0,087^*^ | -0,028 | -0,145^**^ | -0,127^**^ | -0,094^*^ | -0,111^**^ | -0,154^**^ | -0,143^**^ | 0,122^**^ | 0,141^**^ | 0,124^**^ | 0,081^*^ | 1,000 |  |  |  |  |  |
| 25. Bedtime hour | | | 0,236^**^ | 0,053 | 0,057 | 0,105^*^ | | | 0,043 | 0,064 | -0,086 | 0,068 | 0,021 | 0,087^*^ | 0,011 | 0,031 | 0,052 | 0,015 | 0,061 | 0,047 | 0,090^*^ | 0,029 | 0,047 | -0,014 | -0,034 | -0,006 | -0,007 | 0,220^**^ | 1,000 |  |  |  |  |
| 26. Wake after sleep onset | | | -0,219^**^ | -0,027 | -0,056 | -0,018 | | | -0,107^**^ | 0,006 | 0,093 | 0,165^**^ | 0,247^**^ | -0,128^**^ | 0,292^**^ | 0,235^**^ | 0,191^**^ | 0,155^**^ | 0,146^**^ | 0,151^**^ | 0,143^**^ | 0,121^**^ | 0,163^**^ | -0,030 | -0,051 | -0,033 | -0,015 | -0,155^**^ | -0,013 | 1,000 |  |  |  |
| 27.Night awakening’s | | | **-0,391^**^** | 0,001 | 0,010 | 0,021 | | | -0,024 | -0,048 | -0,026 | 0,035 | 0,130^**^ | -0,195^**^ | 0,224^**^ | 0,126^**^ | 0,089^*^ | 0,105^**^ | 0,098^*^ | 0,101^*^ | 0,108^**^ | 0,112^**^ | 0,114^**^ | -0,050 | -0,075 | -0,019 | -0,045 | -0,160^**^ | -0,084^*^ | **0,333^**^** | 1,000 |  |  |
| 28.Poor sleeper | | | -0,098^*^ | -0,106^**^ | 0,042 | 0,026 | | | -0,040 | -0,002 | 0,064 | 0,105^*^ | 0,157^**^ | -0,106^**^ | 0,158^**^ | 0,184^**^ | 0,147^**^ | 0,117^**^ | 0,112^**^ | 0,130^**^ | 0,153^**^ | 0,102^*^ | 0,125^**^ | -0,081^*^ | -0,055 | -0,079 | -0,066 | -0,144^**^ | 0,094^*^ | **0,424^**^** | **0,396^**^** | 1,000 |  |
|  |  |  |  |  |  |  |  | Spearman’s Rho Correlation analyse, * Correlation is significant at the 0.05 level, ** Correlation is significant at the 0.01 level (2-tailed). | | | | | | | | | | | | | | | | | | | | | | | | | |
